# AP1 Transcription Factors in Epidermal Differentiation and Skin Cancer

**DOI:** 10.1155/2013/537028

**Published:** 2013-05-23

**Authors:** Richard L. Eckert, Gautam Adhikary, Christina A. Young, Ralph Jans, James F. Crish, Wen Xu, Ellen A. Rorke

**Affiliations:** ^1^Department of Biochemistry and Molecular Biology, University of Maryland, School of Medicine, 108 North Greene Street, Rm 103, Baltimore, MD 21201, USA; ^2^Department of Dermatology, University of Maryland, School of Medicine, Baltimore, MD 21201, USA; ^3^Department of Obstetrics and Genecology and Reproductive Sciences, University of Maryland, School of Medicine, Baltimore, MD 21201, USA; ^4^Department of Cell Biology, Cleveland Clinic Foundation, Cleveland, OH 44106, USA; ^5^Department of Microbiology and Immunology, University of Maryland, School of Medicine, Baltimore, MD 21201, USA

## Abstract

AP1 (jun/fos) transcription factors (*c-jun, junB, junD, c-fos, FosB, Fra-1, and Fra-2*) are key regulators of epidermal keratinocyte survival and differentiation and important drivers of cancer development. Understanding the role of these factors in epidermis is complicated by the fact that each protein is expressed, at different levels, in multiple cells layers in differentiating epidermis, and because AP1 transcription factors regulate competing processes (i.e., proliferation, apoptosis, and differentiation). Various *in vivo* genetic approaches have been used to study these proteins including targeted and conditional knockdown, overexpression, and expression of dominant-negative inactivating AP1 transcription factors in epidermis. Taken together, these studies suggest that individual AP1 transcription factors have different functions in the epidermis and in cancer development and that altering AP1 transcription factor function in the basal versus suprabasal layers differentially influences the epidermal differentiation response and disease and cancer development.

## 1. Introduction

Keratinocytes are the major cell type responsible for the structure of the epidermis. They begin as stem cells in the basal epidermal layer and hair follicles [1–3]. During differentiation, as the cells migrate to the surface, cell division ceases and morphological changes ensue to produce the spinous, granular, transition, and cornified layers. Spinous layer cells are distinguished by the presence of desmosomal connections, whereas granular layer cells are characterized by the presence of granules that contain the products of keratinocyte differentiation. Differentiation of the granular layer cells results in the formation of the transition zone which separates the dead from living epidermal layers. It is in this zone that the cellular constituents are extensively enzymatically remodeled. This remodeling results in the covalent crosslinking of proteins to produce terminally differentiated corneocytes that form the skin surface [4, 5]. Achieving these morphological alterations relies on executing a preset program of differentiation that requires tight regulation of gene transcription [6].

 The process of activation and suppression of gene transcription is controlled by a diverse family of regulators called transcription factors. Transcription factors mediate the final steps in the relay of information from the cell surface to the nucleus and the gene. This is accomplished by the interaction of the transcription factor with specific DNA elements that are usually located immediately upstream of the sequence that encodes the gene. DNA elements are generally a short DNA sequence of 8–20 nucleotides that encode a specific consensus sequence. A host of transcription factors has been implicated in control of epidermal differentiation and function, including activator protein 1 (AP1), AP2, Sp1, POU domain proteins, and CCAAT enhancer binding proteins [7]. AP1 transcription factors are among the most interesting and important regulators in epidermis [7]. Members of this family (c-fos, fosB, Fra-1, Fra-2, c-jun, junB, and junD) are expressed in specific epidermal layers and control multiple key functions [8]. This review focuses on summarizing interesting animal-based studies designed to identify the impact of perturbing AP1 transcription factor function on epidermal homeostasis and cancer.

## 2. MAPK and AP1 Transcription Factors Are Key Regulators of Keratinocyte Differentiation

**Figure 1:**
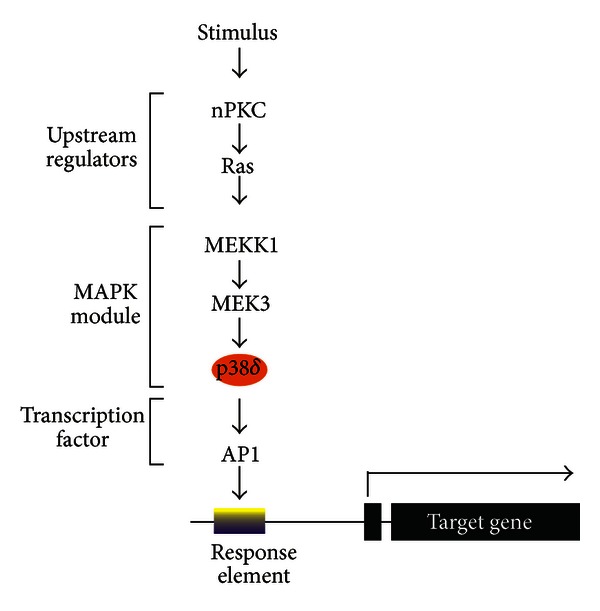
MAPK and AP1 transcription factor control of gene expression. The p38*δ* MAPK cascade that controls the expression of differentiation-associated genes in epidermis is depicted [10]. The three kinases of the MAPK module include MEKK1, MEK3, and p38*δ* MAPK. A differentiation stimulus activates upstream regulatory proteins, in this case novel protein kinase c (nPKC) and the Ras small GTPase. These events lead to phosphorylation and activation of MEKK1 which phosphorylates MEK3 which phosphorylates p38*δ* MAPK. Ultimately p38*δ* MAPK increases AP1 transcription factor expression and activity and the AP1 transcription factors bind to the response element on the target gene promoter to increase transcription.

 The mitogen-activated protein kinases (MAPK) comprise major signaling cascades that regulate differentiation-associated gene expression in epidermis [9–14]. Each MAPK cascade consists of three kinase modulates which include an MEK kinase (MEKK), a mitogen-activate protein kinase/extracellular signal regulated kinase (MEK), and a mitogen-activated protein kinase (MAPK) [15–18]. Activated MEKK phosphorylates MEK which phosphorylates the MAPK. Activated MAPKs phosphorylate a variety of target proteins including transcription factors [10, 19–21]. The most extensively studied MAPKs are the ERK kinases (ERK1, ERK2), the c-jun N-terminal kinases (JNK1, JNK2), and the p38 kinases (p38*α*, *β*, *δ*, and *γ*).  Figure 1 presents a schematic of the p38*δ* MAPK pathway which regulates expression of differentiation-associated genes during keratinocyte differentiation [7, 11]. The cascade consists of upstream regulator proteins (novel protein kinase c and Ras), an MAPK module (MEKK1, MEK3, and p38*δ*) and AP1 transcription factors. Activation of this cascade by a differentiation stimulus causes sequential phosphorylation and activation of kinases in the MAPK module which leads to increased AP1 transcription factor level and binding to the DNA response element in the target gene. This leads to increased target gene transcription [10–14, 22].

 AP1 transcription factors are key downstream targets of MAPK signaling in keratinocytes [12–14, 22–24]. Activator protein one (AP1) transcription factors include jun (c-jun, junB, junD) and fos (c-fos, FosB, Fra-1, Fra-2) family members [25–28]. They form jun-jun and jun-fos dimers that interact with specific AP1 transcription factor consensus DNA binding elements in target genes to regulate expression. They control keratinocyte proliferation [29–31], differentiation [10, 11, 32], and apoptosis [23, 33] and are important in tumor progression and disease development [9–11, 14, 22, 23, 34–38]. As an example, increased p38*δ* MAPK activity results in increased AP1 transcription factor level, increased AP1 transcription factor binding to DNA elements on the involucrin promoter, and increased involucrin gene transcription via a scheme similar to that shown in Figure 1 [8, 39]. The major AP1 factors that interact with the promoter are JunB, JunD, and Fra-1. Moreover, TAM67, a dominant-negative mutant of c-jun that inhibits the activity of all AP1 transcription factors [40], inhibits p38*δ*-dependent involucrin promoter activation [13]. MAPK activation by p38*δ* also results in increased C/EBP*α* and Sp1 binding to DNA binding sites in the involucrin gene promoter [41–43]. Thus, a PKC, Ras, MEKK1, MEK3 pathway activates p38*δ* MAPK and p38*δ*, in turn, acts to increase binding of selected AP1, Sp1, and C/EBP factors to the hINV promoter to increase promoter activity. However, the AP1 transcription factors are the most important family of regulators. In fact, it would be difficult to envision a more important family of transcriptional regulatory proteins in epidermal keratinocytes.

 AP1 action in epidermis is complicated for several reasons. First, multiple AP1 family members are expressed in epidermis and form multiple dimer pairs. AP1 transcription factors can theoretically form eighteen different homo- and heterodimers, and work in other systems show that the particular dimer that is formed influences activity. For example, coexpression of c-fos with c-jun, leading to c-fos:c-jun dimer formation, enhances the transforming capacity of c-jun, whereas pairing c-jun with junB inhibits c-jun transforming capacity [44–46]. These differences may be related to the higher DNA binding and transcriptional activity of c-jun:c-fos heterodimer in comparison to c-jun:junB heterodimer [47]. Thus, it is safe to assume that the dimer that is formed influences activity in differentiating keratinocytes. Second, the expression level of most AP1 family members changes during keratinocyte differentiation [8, 48]. This means that different pairing combinations exist in the basal versus suprabasal layers and that this is likely to drive differences in activity and target gene selection. Third, covalent modification of individual AP1 transcription factors (e.g., phosphorylation) influences activity [49, 50]. For example, c-jun undergoes transient N-terminal phosphorylation as cells exit the G2 phase of the cell cycle, and this state is maintained until the cells complete mitosis [45]. An important lesson from these studies is that the composition of AP1 transcription factors in the tissue and the posttranslational modification state can influence biological activity. The fact that each AP1 transcription factor forms multiple hetero- and homodimers indicates that manipulating the level of one AP1 transcription factor, either by overexpression or knockout, will modify the function of other members. These features must be considered when interpreting the results of studies that alter AP1 transcription factor level or function in epidermis.

## 3. Animal Models of AP1 Transcription Factor Function

 A number of laboratories have used *in vivo* mouse genetic models to study AP1 transcription factor function [34, 51, 52, 52–55]. These include embryonic knockout [54, 56–64], conditional knockout, inducible knockdown, expression of mutant dominant-negative AP1 proteins [65, 65–71], and targeted expression of intact wild-type proteins [72–74, 74–76]. These studies have targeted a variety of tissues, including the epidermis, liver, mammary gland, heart, bone, and blood [77]. The first lesson from these studies is that appropriate AP1 transcription factor expression is required for survival. For example, c-jun knockout mice die at embryonic day E13 due to defects in liver and heart development [78]. Likewise, junB null mice display extraembryonic tissue defects and die at embryonic E9.5 [56]. Fra-1 null mice survive only till embryonic day E9.5, and death is associated with defects in the yolk sac and placenta [54]. JunD knockout mice are born but fail to reproduce due to defects in spermatogenesis and reproduction [64]. These studies indicate that AP1 factors are essential for embryonic survival and are necessary for sustained development and reproduction. This is consistent with a central role for this family of proteins in maintaining tissue and organ homeostasis [77].

 AP1 transcription factors also have tissue-specific effects. An *in vivo* example of this is that transgenic re-expression of junB in junB-null embryos rescues the mice from embryonic death. This is associated with normalization of most tissues; however, the junB transgene is silenced by an epigenetic mechanism in the myeloid lineage, and so these mice develop progressive myeloid leukemia [79]. This is also true in the context of tumor formation where AP1 transcription factors can function as oncogenes or tumor suppressors. For example, junD promotes cell survival by protecting cells from p53-dependent senescence and apoptosis [80, 81]. In contrast, JunD can also antagonize ras-mediated transformation [82]. Fra-1 has a complex role in that it enhances breast cancer cell chemosensitivity by driving cancer stem cells from dormancy [83]. In addition, Fra-1 deficient embryonic fibroblasts are resistant to peroxide-induced cell death, presumably because Fra-1 attenuates Nrf2-driven antioxidant responses [84]. Moreover, Fra-1 is increased in breast cancer where it functions as an oncogene to enhance tumor cell migration [85]. Thus, Fra-1 has multiple roles depending upon the tumor type and conditions.

## 4. AP1 Transcription Factors in Epidermis Knockout and Overexpression Studies

### 4.1. c-Jun and JunB—an Epidermal Oncogene and a Tumor Suppressor

Altering AP1 transcription factor expression changes epidermal function. Mice in which c-jun is conditionally knocked out in the epidermis develop normal skin, but epidermal growth factor receptor (EGFR) level is reduced in the eyelids leading to open eyes at birth [86]. This mimics the phenotype observed in EGFR- or TNF*α*-null mice [87–90]. In addition, in the absence of c-jun, the tumor-prone K5-SOS-F transgenic mice develop smaller epidermal papilloma, suggesting that c-jun is required for tumor formation [86], and it has been noted that c-jun expression is increased in tumors, and overexpression of c-jun in an oncogenic Ras background enhances tumor formation [91]. These findings suggest that c-jun functions as an oncogene in keratinocytes.

 Mice lacking junB in keratinocytes are born with a normal epidermis. However, the epidermis is not completely normal, as epidermal JunB knockout mice display delayed wound healing [51] and develop systemic lupus erythematosus, an autoimmune disease that influences multiple tissues [92]. This phenotype is associated with increased secretion of epidermis-produced interleukin 6 (IL-6) that is associated with loss of JunB-dependent suppression IL-6 gene expression. IL-6 appears to play an essential role in phenotype development, as the phenotype is alleviated when epidermal JunB-null mice are bred to IL-6 deficient mice [92]. Absence of JunB in the epidermis also results in the release of large quantities of epidermis-derived granulocyte-colony stimulating factor (G-CSF) which is associated with skin ulceration, myeloproliferative disease, and low bone mass [93]. G-CSF appears to be essential for phenotype appearance, as breeding JunB null mice into a G-CSF null background reverses the myeloproliferative phenotype [93]. In addition, simultaneous conditional deletion of c-jun and JunB in the epidermis produces a psoriasis-like phenotype [94]. This is associated with increased production of tumor necrosis factor-alpha (TNF*α*) and increased epidermal S100A8/S100A9 expression [52]. Chemokine/cytokine production in epidermis presumably recruits immune cells to the epidermis to produce the psoriatic phenotype. Tissue inhibitor of metalloproteinase-3 (TIMP3) level is reduced in junB/c-jun null epidermis. As TIMP3 is an inhibitor of TNF*α* converting enzyme (TACE), loss of TIMP3 leads to enhanced epidermal TNF*α* cleavage and release [95]. TNF*α* is a key regulator in this context, as the biological phenotype can be mitigated by breeding these mice into a TNF*α*-null background [95]. Moreover, vascular endothelial growth factor (VEGF) also influences this phenotype, as anti-VEGF antibody treated junB/c-jun null mice show a pronounced reduction of inflammatory cells within the dermis and more normal epidermal differentiation [94]. JunB absence also increases tumor forming potential [91]. Tumor formation in Ras-activated cancer cells is inhibited by overexpression of JunB, an effect that requires the JunB transactivation domain [91]. Moreover, expression of dominant-negative JunB in this model, which inhibits JunB function, increases tumor formation [91].

### 4.2. c-Fos Acts as an Oncogene in Epidermis

 JunB and c-jun are the most heavily studied AP1 transcription factors, but information is also available regarding the role of c-fos. Challenge of v-H-ras positive mice with DMBA (7,12-dimethylbenz[a]anthracene) and TPA (12-O-tetradecanoylphorbol-13-acetate), in the two-stage carcinogenesis protocol, increases skin tumor formation. However, tumor formation is attenuated in the absence of c-fos [34] which is associated with increased p53 expression [96]. The higher than normal level of p53 leads to epidermal tumor cell differentiation and suppression of skin tumor formation, in part due to p53-dependent transcriptional activation of TNF*α* converting enzyme [96].

### 4.3. Activating Transcription Factor 2 (ATF2) Suppresses Skin Tumor Formation

Activating transcription factor 2 (ATF2) is a stress-regulated transcription factor, and ATF2 transcriptional activity requires leucine zipper-dependent heterodimerization with members of the AP1 family, including c-jun [97, 98]. Expression of an inactive mutant form of ATF2 (lacking the DNA binding and leucine zipper domains) in the basal epidermis results in reduced tumor formation. When subjected to a two-stage DMBA/TPA skin carcinogenesis protocol, mice expressing the inactive ATF2 display increased tumor formation, and keratinocytes derived from these mice display enhanced anchorage-independent growth [99]. The resulting tumors display enhanced *β*-catenin and cyclin D1 and reduced Notch1 expression. This is consistent with the observation of reduced ATF2 and increased *β*-catenin in human squamous and basal cell carcinoma samples [99] and suggests that ATF2 suppresses epidermal carcinogenesis.

## 5. AP1 Transcription Factors in Epidermis-Dominant-Negative c-Jun (TAM67)

 We have hypothesized that AP1 transcription factors perform different functions in the basal (proliferating) versus suprabasal (differentiating) epidermis [11]. However, testing this hypothesis is complicated by the fact that virtually all of the AP1 family members are expressed, at some level, in both the basal and suprabasal compartments [8, 25, 48]. Thus, we sought a model system where we could achieve complete suppression of AP1 transcription factor function in specific epidermal layers. This goal is difficult to achieve using gene knockout strategies, since knockout normally obviates expression of the targeted gene in all epidermal layers. Thus, we turned to targeted expression of dominant-negative c-jun (TAM67) in specific epidermal layers. In our case, we targeted TAM67 expression to the upper epidermal layers to achieve inactivation of AP1 transcription factor function in the suprabasal epidermis [66]. These studies follow a strategy developed by Nancy Colburn and associates where they targeted TAM67 to the basal epidermal layers using the K14 promoter [100]. This strategy has several advantages. First, TAM67 interferes with the function of all AP1 transcription factors [100]. TAM67 forms heterodimers with other AP1 transcription factors and these complexes bind to DNA, but the complexes are not able to activate transcription [100, 101]. Moreover, an early study, using a keratin promoter to drive expression, showed that TAM67 expression reduces TPA-stimulated invasion of mouse 308 cells through matrigel [65]. Further studies show that TAM67 inhibits invasion of human papillomavirus-immortalized human keratinocytes by suppressing AP1 transcription factor and NF*κ*B signaling [102, 103]. These studies suggest that TAM67 is a useful construct for the study of cell function. Second, our use of the involucrin promoter permits targeting of TAM67 to the suprabasal epidermis [104–106] and alleviates problems that are observed with knockout mice where a specific AP1 transcription factor protein is lost from all layers. Third, a basal layer TAM67-targeted mouse model already existed [68, 70, 71, 107, 108] which permitted a direct comparison of the impact of basal versus suprabasal AP1 transcription factor inactivation. We will first discuss the impact of targeted expression of TAM67 in the epidermal basal layer.

### 5.1. TAM67 in the Basal Epidermis

*In vivo* studies in mouse epidermis show that TAM67-dependent inactivation of AP1 transcription factor function in the basal epidermal layer does not produce obvious changes in keratinocyte proliferation or epidermal or dermal appearance [68, 71, 107]. However, basal layer TAM67 expression does reduce susceptibility of SKH-1 hairless mice to UVB-dependent cancer progression [68, 71, 107]. Both tumor number and size are reduced and this is associated with reduced numbers of cyclin D1 positive cells in the tumors [107]. Expression of the E7 gene from human papillomavirus type 16 in mouse skin induces hyperplasia and enhances tumor promotion, and TAM67 protects mice from E7-enhanced tumorigenesis [70].

 Some additional details are known regarding the mechanism of impact of AP1 transcription factor inaction in epidermal cancer cells. TPA treatment induces transformation of JB6/P+ cells. JB6/P+ cells are murine keratinocytes that undergo transformation following treatment with 12-O-tetradecanoylphorbol-13-acetate (TPA) [109]. Screening of microarrays from TPA-treated JB6/P+ cells, maintained in the presence or absence of TAM67 expression, revealed that high-mobility group A1 (HMGA1) protein is induced by TPA, and this induction is inhibited by TAM67. Further studies show that knockdown of HMGA1 with siRNA reduces JB6/P+ transformation, which is consistent with HMGA1 being an important AP1 transcription factor target [109]. A similar approach, also using JB6/P+ cells, identified sulfiredoxin as an additional gene that is required for TPA-induced transformation and is suppressed by TAM67 [110]. Sulfiredoxin is important for redox homeostasis and acts to reduce hyperoxidized peroxiredoxins. Cyclooxygenase-2, osteopontin, programmed cell death-4, and Wnt5a are additional proteins that may be important in transformation and have been identified [108, 111, 112]. It is possible that these proteins play a role in reducing tumor formation observed in mice where TAM67 is expressed in the basal layer.

### 5.2. TAM67 in the Suprabasal Epidermis

A recent study shows that targeted expression of TAM67 in the suprabasal epidermis results in extensive hyperplasia and hyperkeratosis [66]. This is associated with a substantial increase in proliferation of basal layer keratinocytes as measured by increased BrdU incorporation and increased appearance of Ki67-positive cells. This is not due to a direct effect of TAM67 on basal cells, as two different staining methods reveal that the TAM67-FLAG expression is confined to the suprabasal layers. Thus, inactivating suprabasal AP1 transcription factor function appears to feedback on the basal layer in a manner that stimulates basal layer cell division. In addition, differentiation appears to be delayed and incomplete. Consistent with delayed differentiation, keratins K5 and K14, which are normally exclusively expressed in the basal layer, are detected in all epidermal layers, and K6 is expressed in all epidermal layers. K6 is a keratin that is expressed under conditions of hyperproliferation but is not expressed in normal epidermis [66]. Thus, suprabasal TAM67 expression leads to increased basal layer proliferation and delayed differentiation and ultimately results in extensive hyperkeratosis. This is in marked contrast to the finding that targeting TAM67 to the epidermal basal layer using the keratin 14 promoter (K14-TAM67) produces no overt phenotype under resting conditions [71]. We propose that normal differentiation leads to accumulation of signals, generated by suprabasal cells, that suppress basal layer cell proliferation and that inhibiting differentiation opens this feedback loop leading to increased basal keratinocyte proliferation [66].

 Because of the hyperproliferative phenotype, it was anticipated that mice expressing TAM67 in the suprabasal epidermis would be more susceptible to tumor formation. This was tested by treating control and suprabasal TAM67 mice with a DNA mutagenic agent, 7,12-dimethylbenz[*α*]anthracene (DMBA) to produce initiated cells, and then inducing TAM67 expression. Surprisingly, TAM67 expression, and the associated increase in cell proliferation, did not drive tumor formation in DMBA treated mice. This is interesting, because cell proliferation is thought to predispose tissue to enhanced tumor formation [113]. Treatment with carcinogen (7,12-dimethylbenz[*α*]anthracene, DMBA) followed by tumor promoter (12-O-tetradecanoylphorbol-13-acetate, TPA) is known to cause tumor formation [113]. However, in a protocol where mice were treated with DMBA, followed by treatment with TPA, TAM67 expression reduced tumor formation. The possibility that TAM67 may interfere with the proliferation promoting activity of TPA in the carcinogenesis protocol was considered; however, these experiments suggest that TAM67-expressing epidermis is fully competent to respond to TPA. Taken together, these findings show that inaction of AP1 transcription factor function in the suprabasal epidermis increases epidermal proliferation but reduces carcinogen/tumor promoter-induced cancer development. The underlying mechanism responsible for these surprising observations is under study.

 Thus, although the basal and suprabasal targeted TAM67 mice produce very different epidermal phenotypes, these mice share features in common [66, 71]. First, TAM67 basal and suprabasal epidermal mice respond to stress agents (okadaic acid, TPA, etc.) with increased basal cell proliferation, and this response is not reduced when compared to control mice. Second, both strains display a reduced sensitivity to DMBA/TPA induced tumor formation. The fact that inactivating AP1 factor function in the basal or suprabasal epidermis reduces tumor formation, clearly suggest that, on balance, AP1 factors have an essential role in driving tumor formation.

## 6. Summary

A variety of genetic approaches have been used to study the *in vivo *role of AP1 transcription factors in epidermis. It is clear from these studies that AP1 transcription factors play a key role in controlling differentiation of epidermal keratinocytes and that perturbing this process results in a variety of disease phenotypes including psoriasis and cancer. It is also clear that some AP1 transcription factors function as procancer proteins (e.g., c-jun, c-fos), while others inhibit cancer development (e.g., JunB, ATF2). Additional studies suggest that a host of cytokines and chemokines is involved in generation of the disease and cancer phenotypes that develop when AP1 transcription factor function is perturbed, and these studies suggest that the epidermis can act as an endocrine organ to influence the function of other organs. It also appears that AP1 transcription factors have differing roles in basal and suprabasal epidermis, as inactivation of AP1 transcription factor function in these compartments produces no change (basal targeted TAM67 expression) or hyperproliferation (suprabasal targeted TAM67 expression).
